# Evaluation of serum NEAT1 and MALAT1 expression as diagnostic biomarkers in tyrosinemia, a rare metabolic disorder

**DOI:** 10.1186/s13023-026-04275-9

**Published:** 2026-02-25

**Authors:** Nasrin Motazedian, Mohsen Mohammadi, Negar Azarpira, Kimia Falamarzi, Bita Geramizadeh, Seyed Mohsen Dehghani, Mahdokht Hossein-Aghdaie, Seyed Ali Malekhosseini, Mahintaj Dara

**Affiliations:** 1 Transplant Research Center, Shiraz University of Medical Sciences, Shiraz, Iran; 2 Student Research Committee, Shiraz University of Medical Sciences, Shiraz, Iran; 3 Abu-Ali Sina Organ Transplant Center, Shiraz University of Medical Sciences, Shiraz, Iran; 4 Stem Cells Technology Research Center, Shiraz University of Medical Sciences, Shiraz, Iran

**Keywords:** Tyrosinemia, Long non-coding RNAs, NEAT1, MALAT1, Biomarker

## Abstract

**Objective:**

Tyrosinemia is a rare autosomal recessive inborn error of metabolism caused by a deficiency of fumarylacetoacetate hydrolase (FAH). This leads to the accumulation of toxic metabolites, resulting in progressive liver and kidney damage. This study aimed to evaluate the serum levels of the long non-coding RNAs NEAT1 and MALAT1 in children with tyrosinemia and assess their potential as diagnostic biomarkers.

**Methods:**

This cross-sectional study included 11 children with tyrosinemia, randomly selected from the Shiraz Pediatric Liver Cirrhosis Cohort Study (SPLCCS), and 26 healthy controls. RNA was extracted from serum samples, and the expression levels of NEAT1 and MALAT1 were quantified using quantitative real-time PCR.

**Results:**

Serum NEAT1 expression was significantly upregulated in children with tyrosinemia compared to healthy controls (*p* = 0.011). In contrast, MALAT1 expression showed a non-significant increasing trend. Correlation analysis revealed a positive association between NEAT1 expression and AST and ALP levels, whereas MALAT1 was inversely correlated with INR in the tyrosinemia group. Receiver operating characteristic (ROC) curve analysis demonstrated that NEAT1 has strong diagnostic potential, with an area under the curve (AUC) of 0.945, 100% sensitivity, and 80% specificity at an optimal cut-off value of 1.126. MALAT1 showed poor diagnostic performance.

**Conclusions:**

Our findings suggest that serum NEAT1 represents a promising, non-invasive biomarker for tyrosinemia. In contrast, MALAT1 does not appear to be a useful diagnostic marker for this condition.

**Clinical trial number:**

Not applicable.

## Introduction

Tyrosinemia type 1 is a severe inherited metabolic disorder caused by a deficiency in fumarylacetoacetate hydrolase (FAH), the terminal enzyme in the tyrosine degradation pathway. This defect leads to the accumulation of toxic metabolites, including succinylacetone, which induces hepatic and renal toxicity through mechanisms such as oxidative stress, DNA damage, and the inhibition of key enzymatic activities. Consequently, affected individuals often experience progressive liver failure, renal tubular dysfunction, and an increased risk of hepatocellular carcinoma (HCC) [1]. Without treatment, patients may suffer from recurrent neurological crises, liver cirrhosis, and HCC.

The diagnosis of tyrosinemia relies on a combination of clinical presentation, biochemical findings, and molecular genetic testing [2]. Current management involves treatment with NTBC (Nitisinone), which inhibits the second step of the tyrosine catabolic pathway to prevent the accumulation of toxic metabolites, coupled with a dietary regimen low in tyrosine and phenylalanine. Initiating this treatment early after diagnosis is critical to halt the progression of liver dysfunction and reduce the risk of HCC development. For patients who do not respond to Nitisinone or who present with severe hepatic failure or HCC, liver transplantation remains the definitive therapeutic option [2–4].

Long non-coding RNAs (lncRNAs) are a class of transcripts longer than 200 nucleotides that lack protein-coding potential but play substantial roles in diverse molecular functions. These include the transcriptional and post-transcriptional regulation of gene expression, RNA processing, nuclear organization, and protein modification. A growing body of evidence indicates that lncRNAs are involved in critical biological processes such as cell proliferation, differentiation, organogenesis, and apoptosis, and their dysregulation is increasingly linked to human diseases [5–7]. Notably, emerging research suggests their specific implication in liver pathologies, highlighting their potential as non-invasive biomarkers [5–7].

Nuclear Enriched Abundant Transcript 1 (NEAT1) is a lncRNA located on chromosome 11q13.1 that plays a crucial role in the formation of paraspeckles, nuclear bodies involved in gene regulation and cellular stress responses. NEAT1 is involved not only in physiological processes like organogenesis and immune response but also in the pathogenesis of various cancers, including HCC, cholangiocarcinoma, and gastric cancer. Its upregulation in several malignancies has been associated with patient prognosis [8–10]. Furthermore, evidence points to a role for NEAT1 in the progression of non-alcoholic fatty liver disease (NAFLD), liver fibrosis, and HCC, underscoring its potential as a diagnostic and prognostic biomarker for liver diseases [11–13]. Recent studies have also elucidated its regulatory functions in diverse liver injury states, including sepsis-induced damage, fulminant hepatic failure, and hepatic ischemia-reperfusion injury [14–17].

Metastasis-associated lung adenocarcinoma transcript 1 (MALAT1), also known as NEAT2, is another extensively studied lncRNA located near the NEAT1 locus on chromosome 11q13.1. Although NEAT1 and MALAT1 are transcribed from adjacent regions and show some genomic co-localization, they exhibit distinct binding patterns, suggesting independent yet complementary functions. MALAT1 has been reported to play pivotal roles in central nervous system development, skeletal myogenesis, and vascular growth [18–20]. This lncRNA is also strongly linked to tumorigenesis and cancer cell proliferation in numerous malignancies, including colorectal, gastric, and breast cancers, as well as HCC [18–21]. Accumulating evidence indicates that MALAT1 is critically involved in liver regeneration, NAFLD, liver fibrosis, and cancer [18–21].

Given the severe consequences of tyrosinemia, early diagnosis and effective screening with sensitive biomarkers are of great importance for successful intervention. Furthermore, a deeper understanding of its underlying pathological mechanisms is essential. In this study, we aimed to measure the serum expression levels of the lncRNAs NEAT1 and MALAT1 in children with tyrosinemia and to evaluate their potential for discriminating patients from healthy controls, thereby assessing their utility as diagnostic biomarkers.

## Subjects and methods

### Participants

This cross-sectional study was conducted within the framework of the Shiraz Pediatric Liver Cirrhosis Cohort Study (SPLCCS). The SPLCCS was established in September 2018 by the Shiraz Transplant Research Center to investigate pediatric end-stage liver disease, following approval from the ethics committee of Shiraz University of Medical Sciences (IR.SUMS.REC.1398.142). Written informed consent was obtained from the parents or legal guardians of all participants.

The study cohort consisted of 11 children diagnosed with tyrosinemia, selected from the SPLCCS registry (IR.SUMS.REC.1399.530) [22]. Given the rarity of tyrosinemia and the exploratory (pilot) nature of this first investigation into serum lncRNAs in this condition, a formal prospective sample size calculation was not feasible. Our sample size was determined based on two practical considerations: (1) the availability of all eligible patients from our tertiary national referral center during the study period, and (2) its alignment with sample sizes commonly reported in pilot biomarker studies for rare inborn errors of metabolism. For instance, similar studies have included cohorts of comparable size, such as **12 patients with tyrosinemia type 1** [23] **and 6 patients with Wilson disease** [24].

A control group of 26 healthy children with no history of liver disease was also enrolled. Control participants were recruited from children scheduled for elective minor surgeries (e.g., tonsillectomy). Recruiting perfectly age-matched healthy controls was challenging due to the ethical and practical constraints of performing phlebotomy in very young, healthy children without a medical indicatio**n.** Written informed consent was obtained from their parents or guardians prior to inclusion. The ethical approval for this specific biomarker study was granted by the Ethics Committee of Shiraz University of Medical Sciences (IR.SUMS.MED.REC.1401.456).

### RNA extraction and quantitative real-time PCR (qRT-PCR)

To evaluate circulating lncRNA levels, total RNA was isolated from the serum samples of both patients and controls using RNX-Plus reagent (Cinnagen, Iran), according to the manufacturer’s instructions. Complementary DNA (cDNA) was synthesized from the extracted RNA using the AddScript cDNA Synthesis Kit (Addbio, Korea).

Quantitative real-time PCR amplification was performed on a Step-One ABI instrument (Applied Biosystems, USA) with the following program: an initial denaturation at 95 °C for 10 min, followed by 40 cycles of denaturation at 95 °C for 10 s and a combined annealing/extension step at 62 °C for 60 s. The expression levels of the target lncRNAs, NEAT1 and MALAT1, were normalized to a reference gene and quantified using the 2^-ΔΔCt^ method. All samples were run in triplicate, and mean Ct values were used for analysis [25].

### Statistical analysis

For variables that deviated from a normal distribution, descriptive statistics are reported as the median and interquartile range (IQR). Frequencies and proportions were used for categorical data. Data normality was assessed using the Shapiro-Wilk test, along with skewness and kurtosis indices. The Mann-Whitney U test was used to compare non-normally distributed continuous variables between two groups, and the Chi-square test was used for categorical comparisons.

To account for the significant age difference between the patient and control groups, an Analysis of Covariance (ANCOVA) was employed for group comparisons of key laboratory parameters, with age treated as a covariate. Relationships between lncRNA expression levels and clinical variables were analyzed using Spearman’s correlation for continuous variables and the Mann-Whitney test for dichotomous group comparisons.

To evaluate the diagnostic potential of NEAT1 and MALAT1, receiver operating characteristic (ROC) curves were plotted, and the sensitivity, specificity, and area under the curve (AUC) were calculated. All statistical analyses were performed using SPSS (v16, SPSS Inc., USA) and GraphPad Prism (v9), with a p-value < 0.05 considered statistically significant.

## Results

### Characteristics of case and control groups

This case-control study enrolled 11 children with tyrosinemia and 26 healthy individuals. The demographic and clinical characteristics of the participants are summarized in Table 1. The patient cohort comprised 5 females (45.4%) with a median age of 1.4 years (range: 1.1–2.7 years), whereas the control group included 10 females (38.5%) with a median age of 6 years (range: 4.5–7 years). Consanguinity was observed in the families of seven children with tyrosinemia. Two participants in the tyrosinemia group had a history of liver transplantation, and no fatalities were recorded.

**Table 1:** Demographic and laboratory data of tyrosinemia and control groups

Variables	Case (*n* = 11)	Control (*n* = 26)	*P* value
**Age, y, med (Q1-Q3)**	1.4 (1.1, 2.7)	6.0 (4.52, 7.25)	< 0.001
**Gender**, ***n*(%)**			0.728
Male	6 (54.5)	16 (61.5)	
Female	5 (45.5)	10 (38.5)	
**Parents are relative (yes)**	7 (63.6)	-	
First relative	4 (57.1)	-	
**Liver transplantation (yes)**	2(18.2)		
**AST (U/L)**	55 (34, 138)	22.5 (21.0, 29.0)	< 0.001
**ALT (U/L)**	45 (32.7, 84)	7.0 (4.75, 12.0)	< 0.001
**ALKP (U/L)**	974 (639, 1158)	402 (353, 501.1)	< 0.001
**Albumin (g/dL)**	4.1 (3.0, 4.7)	3.9 (3.58, 4.12)	0.612
**Total protein (g/dL)**	6.8 (6.0, 7.68)	5.94 (5.45, 6.45)	0.019
**Total Bill (mg/dL)**	0.71 (0.62, 1.05)	0.4 (0.4, 0.5)	0.004
**Direct Bill (mg/dL)**	0.3 (0.19, 0.50)	0.2 (0.1. 0.24)	0.040
**Hb (g/dL)**	11.8 (11.5, 12.3)	13.0(12.3, 13.9)	0.002
**WBC per 1000**	7.1 (5.3, 14.4)	6.9 (6.0, 8.8)	0.883
**Platelet per 1000**	192 (136, 232)	310.5 (284.2, 356.6)	< 0.001

Statistical comparisons revealed significantly elevated liver enzymes and bilirubin in the tyrosinemia group compared to controls, including AST, ALT, ALP, and total bilirubin (*p* < 0.001 for all). Conversely, the patient group showed significantly decreased hemoglobin levels (*p* = 0.002) and platelet counts (*p* < 0.001).

### Expression of NEAT1 and MALAT1 in serum of case and control groups

**Fig. 1:**
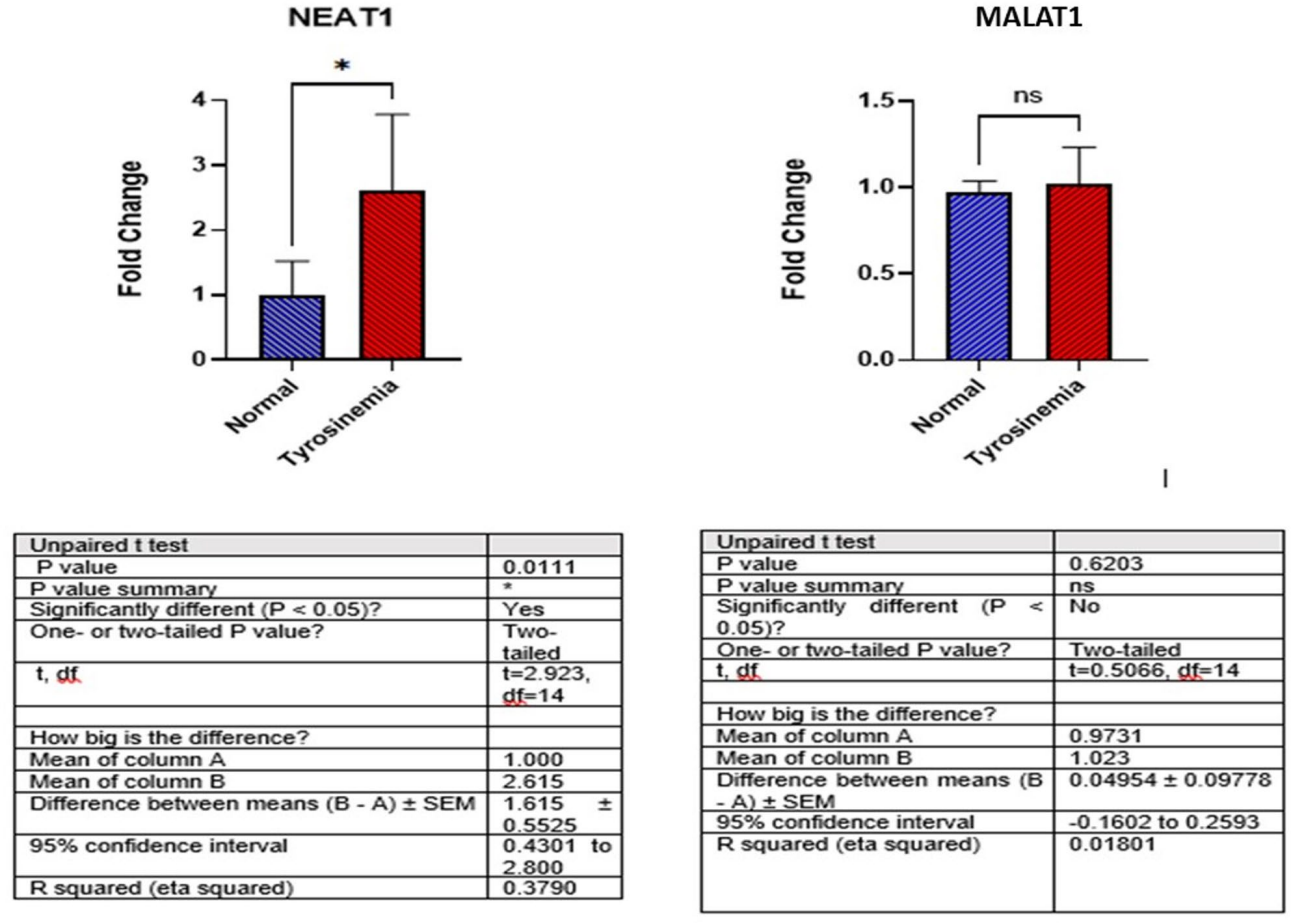
LncRNA NEAT1 and MALAT1 expression levels in children with tyrosinemia and healthy subjects

In this study, we investigated the serum expression levels of the long non-coding RNAs NEAT1 and MALAT1 in children with tyrosinemia compared to healthy controls. Our analysis revealed a significant upregulation of NEAT1 in the tyrosinemia group, with a mean fold change of 2.61 compared to 1.00 in healthy controls (*p* = 0.011). In contrast, although MALAT1 expression showed a slight increase in the tyrosinemia group (mean fold change of 1.02 vs. 0.97 in controls), this difference was not statistically significant (*p* = 0.62) (Fig. 1).

### Association analysis of NEAT1 and MALAT1 expression with clinical and biochemical characteristics of tyrosinemia cases

Further analysis was conducted to explore potential correlations between the expression levels of NEAT1 and MALAT1 and various demographic and clinical variables within the tyrosinemia cohort. No significant associations were found with demographic factors, including gender, parental consanguinity, or history of liver transplantation.

In the analysis of NEAT1, a strong positive correlation was observed with serum AST (*r* = 0.736, *p* = 0.010) and ALP (*r* = 0.659, *p* = 0.027) levels. No other significant correlations were identified between NEAT1 and other biochemical parameters.

Regarding MALAT1, a significant inverse correlation was found with INR (*r* = -0.644, *p* = 0.044). No other significant correlations were observed between MALAT1 expression and other measured laboratory values, including liver enzymes, bilirubin, albumin, hemoglobin, or platelet count. These correlation data are detailed in Tables 2 and 3.

**Table 2:** Relationship between NEAT1 and MALAT1 expression and children with tyrosinemias’ demographic data

	MALAT1		NEAT1	
	Mean ± SD	*P*-value	Mean ± SD	*P*-value
**Gender**				
Male	0.99 ± 0.29	0.637	3.20 ± 1.18	0.059
Female	1.05 ± 0.04		1.90 ± 0.69	
**Parents relevant**				
First relative	1.13 ± 0.31	0.614	2.84 ± 1.15	0.139
Second relative	1.03 ± 0.03		1.55 ± 0.53	
**Liver transplantation**				
Yes	0.94 ± 0.09	0.614	1.86 ± 0.77	0.337
No	1.03 ± 0.22		2.78 ± 1.20	
**Complication (Ascites, esophageal varices)**				
**Yes**	0.96 ± 0.22	0.710	3.53 ± 0.88	0.237
**No**	1.03 ± 0.21		2.41 ± 1.15	

**Table 3:** Correlation between NEAT1 and MALAT1 levels and laboratory findings of tyrosinemia cases

Variables	MALAT1		NEAT1	
	rho	*P*-value	rho	*P*-value
Age (y)	-0.092	0.788	-0.597	0.053
AST (U/L)	-0.457	0.158	0.736	0.010
ALT (U/L)	-0.442	0.174	0.678	0.022
ALKP (U/L)	-0.291	0.385	0.659	0.027
Albumin (g/dL)	0.472	0.143	0.075	0.827
Total protein (g/dL)	-0.062	0.855	0.271	0.419
Total Bill (mg/dL)	-0.342	0.304	0.356	0.282
Direct Bill (mg/dL)	-0.369	0.265	0.129	0.706
INR	-0.644	0.044	0.485	0.156
PT (S)	-0.553	0.097	0.541	0.106
Hb (g/dL)	0.401	0.221	-0.368	0.265
WBC per 1000	0.200	0.556	-0.123	0.719
Platelet per 1000	0.430	0.187	-0.203	0.549

### Diagnostic value of NEAT1 and MALAT1

**Fig. 2:**
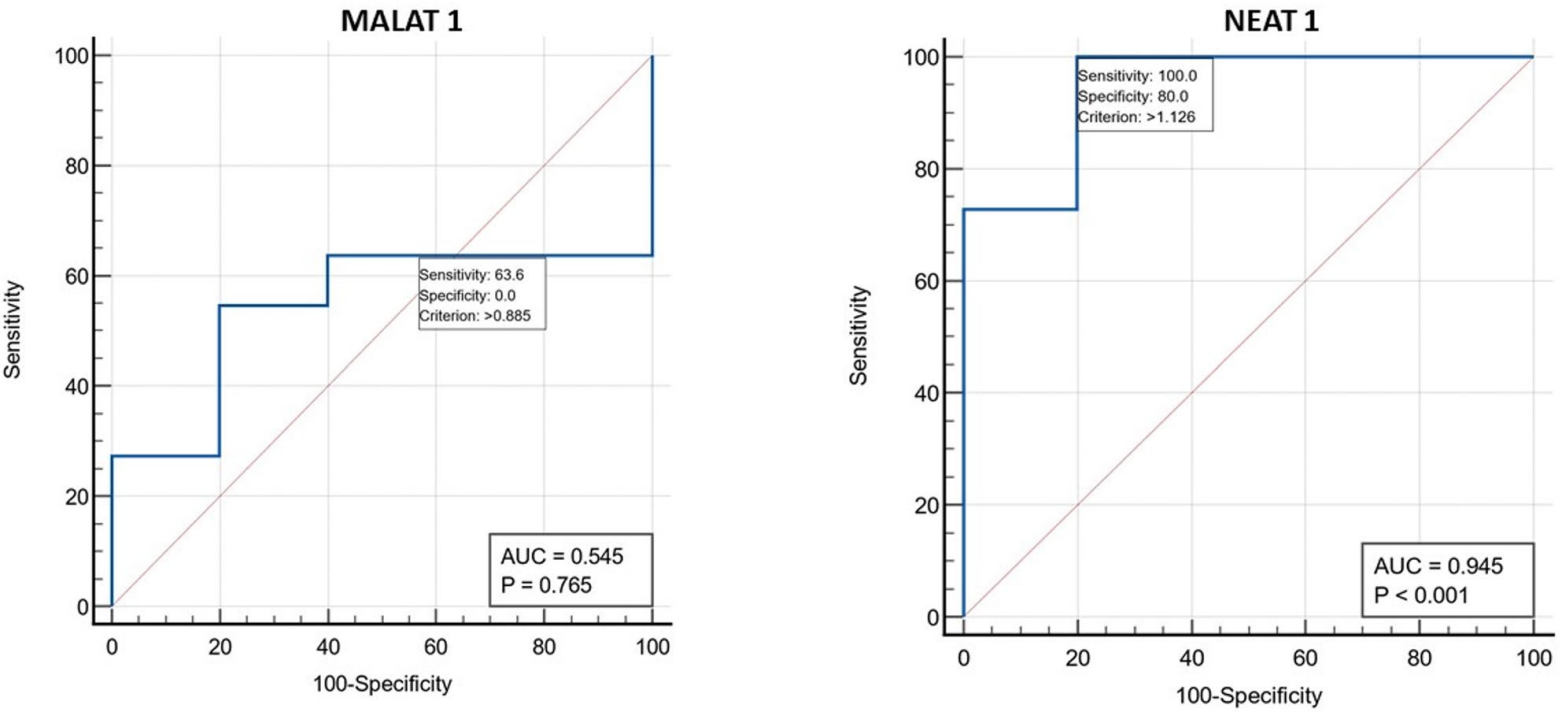
ROC curves for NEAT1 and MALAT1 lncRNAs

The diagnostic potential of NEAT1 and MALAT1 was assessed using receiver operating characteristic (ROC) curve analysis (Fig. 2). NEAT1 demonstrated outstanding diagnostic performance, with an area under the curve (AUC) of 0.945 (*p* < 0.001). At the optimal cut-off value of 1.126, NEAT1 achieved a sensitivity of 100% and a specificity of 80%. In contrast, MALAT1 showed no significant diagnostic utility, with an AUC of 0.545 (*p* = 0.765). At its cut-off value of 0.885, the sensitivity was 63.6%, and it failed to provide any meaningful specificity (0%).

## Discussion

Optimal outcomes for children with tyrosinemia depend on the early initiation of treatment with nitisinone and a low tyrosine/phenylalanine diet [4, 26]. However, even with early treatment, patients remain at risk of developing liver cancer, underscoring the critical need for sensitive biomarkers and a deeper understanding of disease pathogenesis.

Recent investigations have highlighted the association of various long non-coding RNAs (lncRNAs) with liver diseases and their potential as diagnostic and prognostic biomarkers [6, 27]. Therefore, we conducted this study to analyze the expression of NEAT1 and MALAT1 in children with tyrosinemia. Our key finding is a significant upregulation of NEAT1 in the serum of children with tyrosinemia compared to healthy controls. Furthermore, NEAT1 expression showed a strong positive correlation with serum AST and ALP levels. Most notably, ROC curve analysis demonstrated that NEAT1 possesses outstanding diagnostic utility, with an AUC of 0.945, high sensitivity (100%), and good specificity (80%). In contrast, while MALAT1 expression was slightly elevated in the tyrosinemia group, this difference was not statistically significant, and it showed no meaningful diagnostic power. The differential diagnostic performance of NEAT1 and MALAT1 likely reflects their engagement in distinct molecular pathways. We speculate that the abnormal expression of MALAT1 may be mainly associated with liver injury related to viral hepatitis and fatty liver disease, whereas the metabolic liver injury in tyrosinemia does not activate its expression pathway. This is consistent with our finding that MALAT1 was not significantly elevated. Conversely, NEAT1 is known to be upregulated in response to oxidative and metabolic stress hallmarks of tyrosinemia making it a more sensitive biomarker for this specific metabolic disorder. This etiologic specificity underscores the potential clinical relevance of NEAT1 over MALAT1 in tyrosinemia.

Our findings on NEAT1 are consistent with its documented roles in various liver pathologies. Beyond its functions in physiological and other pathological processes, NEAT1 is implicated in the progression of NAFLD, HCC, and liver fibrosis [8]. It has been reported to worsen HCC progression, and targeting it may increase the sensitivity of cancer cells to therapy [28, 29]. Although one study did not detect NEAT1 in the serum or liver tissues of NAFLD patients [11], it has been proposed as a candidate regulator of lipid accumulation, inflammation, and fibrosis in NAFLD [8]. Furthermore, Zhao et al. demonstrated that NEAT1 was significantly increased in HCC tissues and correlated with cirrhosis, TNM stage, and microvascular invasion, suggesting its role as an independent prognostic factor [9]. Recent evidence also points to a crucial role for NEAT1 in acute and chronic liver failure. For instance, Gao et al. reported that serum exosomal NEAT1 was significantly elevated in non-survivors of acute-on-chronic hepatitis B liver failure (ACHBLF), proposing it as a prognostic biomarker superior to the MELD score for predicting 90-day mortality [30]. Elevated NEAT1 has also been observed in the hepatic tissues of patients with sepsis-induced liver injury [16] and in the peripheral blood mononuclear cells of patients with acute-on-chronic liver failure, where it correlated with pro-inflammatory cytokines [15].

LncRNA MALAT1, known for its diverse roles in biological processes and its overexpression in various human diseases [31], has also been studied in liver conditions such as fatty liver disease, liver fibrosis, and HCC [31]. It is significantly increased in HCC tissues, promoting cell proliferation and metastasis [32], and is associated with a greater risk of tumor recurrence post-liver transplantation [33]. In the context of HCV-induced HCC, elevated serum MALAT1 levels correlated with clinical stage and tumor number [34]. While our study found no significant correlation between MALAT1 and liver enzymes in tyrosinemia, other studies in different liver conditions have reported such associations. For example, Toraih et al. found MALAT1 correlated positively with AST and total bilirubin, and inversely with hemoglobin in an HCC cohort [34]. Similarly, MALAT1 was overexpressed in patients with NASH compared to simple steatosis, and its levels correlated positively with ALT, AST, and ALP [35]. The discrepancies between our findings and these studies are likely attributable to differences in cohort sizes, the specific etiology of the liver disease (metabolic vs. viral vs. fatty liver), and the distinct pathological mechanisms of tyrosinemia compared to other hepatic disorders.

### Limitation

This study should be interpreted as a comprehensive, pilot investigation of a rare disease. The subgroup comparisons, particularly between transplanted and non-transplanted children with tyrosinemia, are severely limited by the very small sample size and should be considered hypothesis-generating for future multi-center studies. Furthermore, the age discrepancy with the control group, while a limitation, was an unavoidable consequence of the ethical challenges in sampling healthy infants.

## Conclusion

In conclusion, our results indicate that the expression of lncRNA NEAT1 was significantly elevated in the serum of children with tyrosinemias with tyrosinemia compared to healthy individuals, while the increase in MALAT1 expression was not statistically significant. These findings suggest that NEAT1, in particular, could serve as a novel, non-invasive biomarker for tyrosinemia, warranting further investigation. To the best of our knowledge, this is the first study to assess the expression of these two lncRNAs in tyrosinemia. However, it is crucial to interpret these findings within the context of the study’s limitations, primarily the small sample size, which precludes definitive conclusions, especially regarding MALAT1. Therefore, larger multi-center studies with long-term follow-up are essential to validate these preliminary results, elucidate the exact role of NEAT1 and MALAT1 in tyrosinemia pathology, and determine the clinical prognostic value of their overexpression.

## Data Availability

The data is available upon request from the corresponding authors, Mahintaj Dara or Negar Azarpira.
